# Effectiveness of in-service training plus the collaborative improvement strategy on the quality of routine malaria surveillance data: results of a pilot study in Kayunga District, Uganda

**DOI:** 10.1186/s12936-021-03822-y

**Published:** 2021-06-29

**Authors:** Nelli Westercamp, Sarah G. Staedke, Catherine Maiteki-Sebuguzi, Alex Ndyabakira, John Michael Okiring, Simon P. Kigozi, Grant Dorsey, Edward Broughton, Eleanor Hutchinson, M. Rashad Massoud, Alexander K. Rowe

**Affiliations:** 1 Malaria Branch, Division of Parasitic Diseases and Malaria, Centers for Disease Control and Prevention, 1600 Clifton Road, Atlanta, GA 30333 USA; 2 Department of Clinical Research, London School of Hygiene and Tropical Medicine, Keppel Street, London, WC1E 7HT UK; 3 Infectious Diseases Research Collaboration, 2C Nakasero Hill Road, Kampala, Uganda; 4 Department of Medicine, University of California, San Francisco, USA; 5 ASSIST Project, University Research Co., LLC, 5404 Wisconsin Avenue, Suite 600, Chevy Chase, MD 20815 USA

**Keywords:** Collaborative improvement, Quality improvement, Malaria, Surveillance, Uganda, Data quality

## Abstract

**Background:**

Surveillance data are essential for malaria control, but quality is often poor. The aim of the study was to evaluate the effectiveness of the novel combination of training plus an innovative quality improvement method—collaborative improvement (CI)—on the quality of malaria surveillance data in Uganda.

**Methods:**

The intervention (training plus CI, or TCI), including brief in-service training and CI, was delivered in 5 health facilities (HFs) in Kayunga District from November 2015 to August 2016. HF teams monitored data quality, conducted plan-do-study-act cycles to test changes, attended periodic learning sessions, and received CI coaching. An independent evaluation was conducted to assess data completeness, accuracy, and timeliness. Using an interrupted time series design without a separate control group, data were abstracted from 156,707 outpatient department (OPD) records, laboratory registers, and aggregated monthly reports (MR) for 4 time periods: baseline—12 months, TCI scale-up—5 months; CI implementation—9 months; post-intervention—4 months. Monthly OPD register completeness was measured as the proportion of patient records with a malaria diagnosis with: (1) all data fields completed, and (2) all clinically-relevant fields completed. Accuracy was the relative difference between: (1) number of monthly malaria patients reported in OPD register versus MR, and (2) proportion of positive malaria tests reported in the laboratory register versus MR. Data were analysed with segmented linear regression modelling.

**Results:**

Data completeness increased substantially following TCI. Compared to baseline, all-field completeness increased by 60.1%-points (95% confidence interval [CI]: 46.9–73.2%) at mid-point, and clinically-relevant completeness increased by 61.6%-points (95% CI: 56.6–66.7%). A relative − 57.4%-point (95% confidence interval: − 105.5, − 9.3%) change, indicating an improvement in accuracy of malaria test positivity reporting, but no effect on data accuracy for monthly malaria patients, were observed. Cost per additional malaria patient, for whom complete clinically-relevant data were recorded in the OPD register, was $3.53 (95% confidence interval: $3.03, $4.15).

**Conclusions:**

TCI improved malaria surveillance completeness considerably, with limited impact on accuracy. Although these results are promising, the intervention’s effectiveness should be evaluated in more HFs, with longer follow-up, ideally in a randomized trial, before recommending CI for wide-scale use.

**Supplementary Information:**

The online version contains supplementary material available at 10.1186/s12936-021-03822-y.

## Background

In most African countries, health facility (HF)-based surveillance data on malaria reported through routine health information systems are a critical source of information for disease surveillance and decision-making by national malaria control programmes. Unfortunately, challenges in HF data completeness, accuracy, and timeliness [1–12] limit the utility of routinely collected HF data for programmatic monitoring and evaluation [4, 13, 14]. As a result, modelling has been used to estimate malaria morbidity and mortality trends in many countries [15, 16].

Given these limitations in HF surveillance data, periodic cross-sectional population-based surveys have been considered a superior source of information for planning and policy needs [17]. However, such surveys are complex and costly, are not useful for assessing malaria prevalence trends in low-burden settings, and lack the geographic granularity of HF surveillance data. Due, in part, to the global scale-up of malaria control and elimination efforts, there is an increased demand for near real-time information to track malaria burden and guide implementation of interventions [18, 19]. The widespread adoption of District Health Information System 2 (DHIS2) in African countries has improved the availability of HF data by integrating disease-specific surveillance systems into a single digital platform, rendering the timely use of HF surveillance data more feasible.

The DHIS2 platform was established in Uganda in 2012, and the system has been credited with increasing the quality, availability, and use of national HF data substantially [20, 21]. The malaria-specific goals of DHIS2 in Uganda include collating and presenting data needed to calculate indicators on adherence to test-and-treat guidelines [22], malaria burden, and the targeting and evaluation of interventions. While DHIS2 is envisioned as a digital disease surveillance platform, actual data capture remains a manual process involving thousands of health workers from individual HFs. For malaria, data collection requires paper-based data extraction from paper registers sourced from outpatient departments (OPD), inpatient units, laboratories, and pharmacies. Collected data are aggregated monthly at HFs and submitted, as paper reports, to the district level where information is entered into the web-based DHIS2 [20].

Until recently, HF registers lacked several key data fields required to calculate the indicators outlined in Uganda’s malaria strategic goals. To address this deficiency, the Uganda Ministry of Health (MOH) introduced revised HF registers and reporting forms (heretofore referred to as “revised forms”) in July 2015. In addition to capturing the required malaria-related data, the revised forms include new fields to collect data on tuberculosis burden and care, health behaviour, nutritional status, referral, and selected clinical practices.

These ambitious new data collection plans increased the amount and complexity of data that Ugandan health workers were required to record substantially, doubling the number of data entry fields, including sensitive health behaviour questions, and requiring additional equipment for specific measurements (e.g., for blood pressure and blood sugar levels). While the revised forms may improve the quality and utility of collected data, there also is a substantial risk that data quality could be undermined by the increased complexity and volume of work. Recognizing this risk and seeking to improve the overall quality of DHIS2 data from HFs, the Ugandan MOH and malaria stakeholders sought to identify innovative, feasible, context-appropriate, healthcare worker-centred methods—one of which was the collaborative improvement (CI) approach.

CI was developed by the Institute for Healthcare Improvement in 1995 as a team-based approach to improve healthcare worker performance. Over the last 20 + years, the CI approach has been applied across a wide array of healthcare worker performance areas in high-resource [23–26] and low-resource [26–35] settings. As an adjunct to traditional training modalities, strategies including CI components have been shown to improve results substantially [32, 34]. The CI approach involves a network of teams (usually in HFs) that identify problems, develop solutions, measure change using agreed-upon indicators, and share results and best practices through periodic learning sessions [28, 36, 37]. Specific CI components include the initial design of a change package by national and international technical experts, networking of facility-based CI teams (proof of concept CI networks can include a few HFs, while CIs with an established intervention may involve 20–100 HFs), training or sensitization towards standards, application of continuous quality improvement techniques known as plan-do-study-act cycles to test changes [38, 39], ongoing coaching and mentorship on the quality assurance methodology, and integration of “shared learning” to drive change and promote innovation.

Although the evidence for CI effectiveness is encouraging, much of it was produced by evaluations with important methodological limitations [40, 41], including inadequate control arm, reliance on self-evaluation and self-reported internal monitoring data as sole outcome measures [27, 32, 42–46], and short baseline periods, which limit the ability to fully account for natural variability in performance [27, 32]. Moreover, because the CI process encourages improved data collection, a positive bias can result when under-reporting at baseline is corrected and inaccurately interpreted as improvement [23, 26]. Lastly, the majority of published CI studies are limited to large tertiary and academic centres with limited evaluation of effectiveness in primary (lower) level HFs [33, 47].

To evaluate the feasibility, cost-effectiveness, and impact of a combined in-service training and CI initiative on the quality of malaria surveillance data, a pilot study of five rural Ugandan HFs was conducted using an interrupted time series design, independent evaluation, and objective data sources. The small scale of the pilot allowed a unique in-depth qualitative exploration of the mechanisms of action of CI components [48].

## Methods

The objectives of this study were to estimate the effectiveness and costs of an intervention that combined in-service healthcare worker training plus CI (referred to as TCI), which was designed to improve the quality of routine malaria surveillance data collected at HFs in Uganda.

### Study design

This was a one-arm interrupted time series study, which used baseline trends as a counterfactual, to determine the effectiveness of TCI [49]. A cost-effectiveness evaluation was completed using activity-based costing to calculate the total incremental intervention cost divided by the number of improved records observed during the study period. The study had a 12-month baseline period, 5 months of TCI intervention scale-up, 9 months of active TCI implementation, and 4 months of post-intervention evaluation.

### Study sites

The study was conducted in five hierarchically-connected public HFs (level II, III, and IV) in the Kayunga District, Central Uganda—a moderate-to-high malaria endemic area. Level II health centres (HC IIs) serve ~ 5000 residents and generally have no laboratory facilities (malaria diagnosis by RDT only); level III health centres (HC IIIs) usually serve 20,000 residents and are expected to have a functioning laboratory (malaria diagnosis by RDT and microscopy) and maternity services; and level IV health centres (HC IV) serve a health sub-district (100,000 residents) and are designed to function as a small hospital serving outpatients and inpatients (malaria diagnosis by RDT and microscopy).

Of the 18 HFs in Kayunga District (8 level II HCs, 8 level III HCs, and 2 level IV HCs), two were excluded due to prior experience with CI or current engagement in externally-funded malaria research or data improvement interventions. All other HFs were eligible (Box 1). The final selection was made from HFs within the same hierarchical unit, balanced by HC level representation: Bbaale HC IV, Lugaasa HC III, Wabwo Oko HC III, Nakyesa HC II, and Kakiika HC II. The detailed characteristics of selected facilities are reported separately, in the paper reporting on the results of the qualitative evaluation [48].

Box 1: Health facility eligibility criteria

**Table:** 

Inclusion criteria:	
Located in areas of moderate or high malaria transmission	
Located within geographic proximity to each other, preferably within the same district Public, non-prison, Level II–IV facilities with at least 4 health workers Facility director/in-charge and staff receptive to the intervention District-level administration receptive to the intervention Considered “typical” performing facilities in terms of case management, recording and reporting practices, based on DHIS2 data review and district administration information Outpatient and lab registers, stock and pharmacy records, and monthly reports available for retrospective review for the year prior to study participation. Exclusion criteria: Lack of acceptance or willingness to participate in quality improvement Previous experience with quality improvement collaborative methods	
Current participation in malaria research, malaria programmes, or externally funded interventions for data collection

### Study outcomes

Study outcomes included data completeness, accuracy, and timeliness, defined as follows (Table 1): completeness: the proportion of OPD register malaria records with all fields complete, per month; accuracy: the relative difference in patient counts or malaria tests between the OPD registers, laboratory registers, and submitted monthly reports, per month; timeliness: the proportion of monthly summary reports submitted within 15 days of the end of the month, as requested by the MOH.

**Table 1:** Formulae and data sources for completeness and accuracy outcomes

Outcomes and formulae	Data sources
Completeness outcomes, per month	
Completeness of all field = $\frac{{\# \;{{\rm of}}\;{{\rm malaria}}\;{{\rm records}}^{{{\rm a}}} \;{{\rm with}}\;{{\rm all}}\;{{\rm fields}}\;{{\rm complete}}}}{{\# \;{{\rm malaria}}\;{{\rm records}}}}$	OPD^b^ register
Completeness of clinically relevant fields^c^ = $\frac{{\# \;{{\rm of}}\;{{\rm malaria}}\;{{\rm records}}\;{{\rm with}}\;{{\rm clinically}}\;{{\rm relevant}}\;{{\rm fields}}\;{{\rm complete}}}}{{\# \;{{\rm of}}\;{{\rm malaria}}\;{{\rm records}}}}$	OPD register
Accuracy outcomes (measured as relative differences), per month	
Accuracy of reported malaria cases = $\frac{{\left| {\# \;{{\rm malaria}}\;{{\rm cases}}\;{{\rm in}}\;{{\rm OPD}} - \# {{\rm malaria}}\;{{\rm cases}}\;{{\rm in}}\;{{\rm monthly}}\;{{\rm reports}}} \right|}}{{\# \;{{\rm of}}\;{{\rm malaria}}\;{{\rm cases}}\;{{\rm in}}\;{{\rm OPD}}}}$	OPD register vs. monthly reports
Accuracy of reported TPR^d^ = $\frac{{\left| {{{\rm TPR}}\;{{\rm based}}\;{{\rm on}}\;{{\rm lab}}\;{{\rm register}} - {{\rm TPR}}\;{{\rm based}}\;{{\rm on}}\;{{\rm monthly}}\;{{\rm report}}} \right|}}{{{{\rm TPR}}\;{{\rm based}}\;{{\rm on}}\;{{\rm lab}}\;{{\rm register}}}}$	Lab register vs. monthly reports

^a^Malaria records: an individual-level patient register entry of patients diagnosed with malaria

^b^OPD: outpatient department

^c^Clinically-relevant fields: age, sex, weight, diagnosis, and treatment

^d^TPR: test positivity rate (proportion of all malaria tests done with a positive result)

### Study intervention

**Fig. 1:**
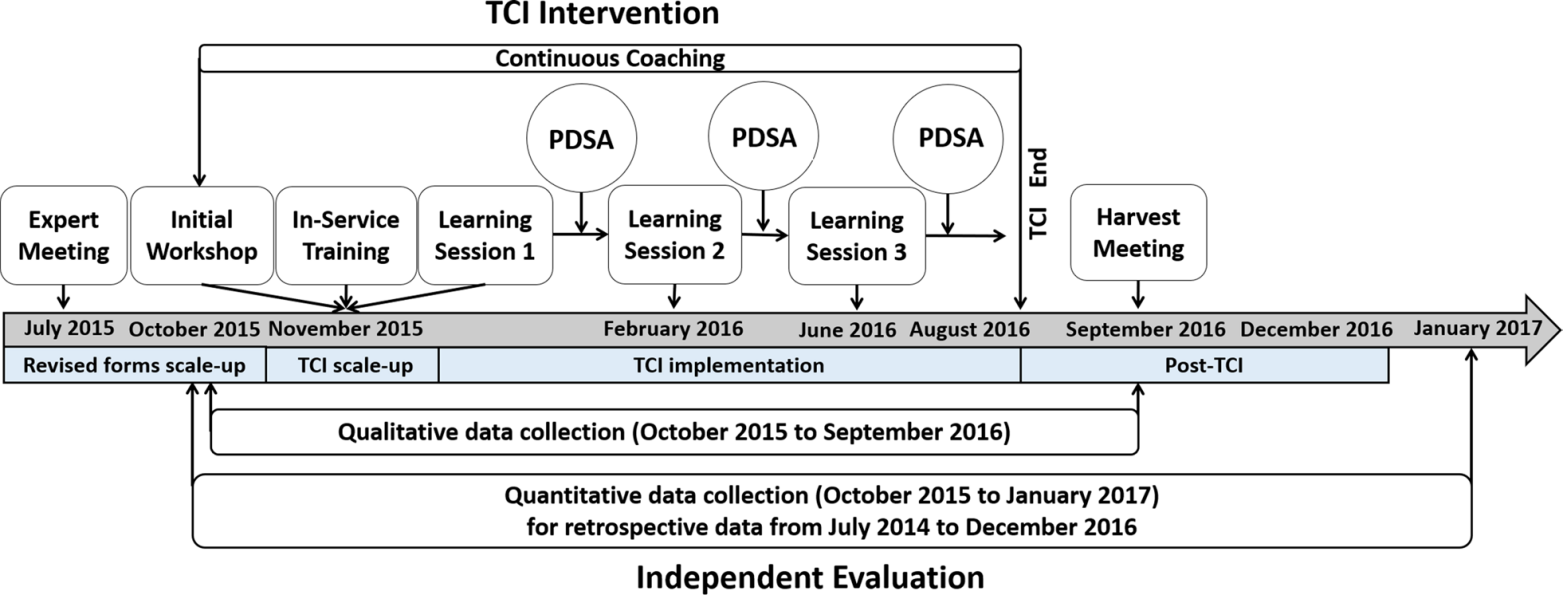
Timing of study procedures. *TCI* in-service training and collaborative improvement, *PDSA* Plan-do-study-act

Prior to implementation, a full-day expert meeting was held to review relevant evidence and define the scope of the collaborative (Fig. 1). Each HF formed a CI team comprised of representatives from relevant departments (e.g., clinicians, lab, pharmacy, records) in preparation for the study activities. The intervention started in November 2015 with an on-site in-service training (3–4 h at each HF) for HF staff on good malaria data recording and reporting practices. Also in November 2015, a half-day workshop was held to orient district and HF administrators to the CI methodology and the study, followed by the first learning session. CI teams attended a series of 1–2 day learning sessions held approximately every 3 months. Learning sessions provided didactic instruction on CI methodology and allowed teams to share their experiences and discuss challenges. CI teams were responsible for recording study outcomes each month, or more frequently if new changes were implemented, and producing graphs of the outcomes over time (run charts) in quality improvement journals supplied by the CI implementers. Summaries of the implemented changes (see Additional file 1: Annex 1 for examples) and the outcomes documented in the journals were presented and discussed during learning sessions, so CI teams could learn from each other which changes were more or less effective. At the end of each learning session, CI teams developed action plan and identified outcomes to focus on. Each learning session was followed by an “action period” during which plan-do-study-act cycles were implemented to test change ideas. Ongoing CI coaching and mentorship was provided to each team throughout the intervention period. The CI mentor was a trained and experienced professional who provided onsite coaching (1–2 h) on the CI methodology rather than the technical aspects, several times per action period.

Each learning session initiated a new intervention phase – for three total phases:

- Phase I (November 2015–February 2016), improving completeness of OPD and lab registers.
- Phase II (March–June 2016), improving concordance between the OPD registers, lab registers, and monthly summary reports.
- Phase III (June–August 2016), harmonizing OPD and lab registers for calculation of standard malaria outcomes.

While the improvements were intended to be cumulative in nature, the CI teams primarily tracked the outcomes reflecting the specific focus of each phase.

In addition to the described outcomes, CI teams also monitored intermediate indicators to assess specific aspects targeted by implemented changes, such as number of malaria patients that can be traced in both OPD and laboratory registers and number of patients with a positive malaria test result in both OPD and laboratory registers. Each team performed independent root cause analyses and identified changes to implement at their health facilities. Aside from the costs associated with training, learning sessions, and coaching, external resources were not provided for changes instituted by the HF CI teams.

A “harvest meeting” was held over two days in September 2016 to complete the TCI intervention. CI teams documented and ranked the most effective changes made by the CI teams, which were compiled into a “change package” (see Additional file 1: Annex 1). This “change package” could be used as a catalogue of changes that improved data quality, which could be adopted by other health centres or by the MOH on a larger scale, considering the local context.

### Evaluation

Independent quantitative and qualitative evaluations were carried out alongside the study intervention (Fig. 1, lower section). Quantitative results are presented in this paper, and qualitative results are published separately [48, 50]. Data were collected from the OPD and lab registers and the monthly summary reports by a team of seven trained surveyors during HF visits between October 2015 and January 2017, for the period from July 2014 to December 2016. Individual level data were double-entered for 20% of the records, and all data were compared to the aggregate data collected separately.

### Data analyses

Monthly data were analysed using segmented linear regression modelling, based on the following timelines:

Segment 1. Baseline: June 2014 to June 2015 (12 months), retrospective data collected from the registers and monthly reports.

Intervention scale-up (revised forms plus TCI; excluded from segmented regression analysis). The scale-up period included: (1) Scale-up of revised forms by the MoH (July to October 2015)*.* The revised forms implemented by the Ugandan MOH were introduced and scaled up over 4 months (July–October 2015), as part of a mandatory nationwide roll-out in July 2015 accompanied by cascade training, although this training was limited in Kayunga district (one health worker per HF); (2) Scale-up of the TCI (November 2015).

Segment 2. Follow-up period (revised forms plus TCI plus post-TCI): December 2015 to December 2016 (13 months), including 9 months of TCI implementation (October 2015 to August 2016) and 4 months of a short-term post-intervention period (September to December 2016).

The segmented linear regression modelling compared the baseline (segment 1) and the follow-up (segment 2) periods to estimate three effect sizes for each outcome:

1. Immediate level change from the end of the baseline period to the immediate period after implementation,
2. Baseline-to-follow-up change in outcome trend, and
3. Single effect size combining the immediate level change and trend change, calculated as the outcome level at the mid-point of the follow-up period, as predicted by the segmented linear regression model minus a predicted counterfactual value based on the baseline trend extended to the mid-point of the follow-up period. The single effect size can be measured as an absolute or a relative difference, the latter being more meaningful for indicators with small values.

Models were adjusted for serial autocorrelation resulting from repeated observations over time through SAS AUTOREG procedure. Statistical analyses were performed using SAS v. 9.3 (SAS Institute Inc., Cary, NC, USA).

The self-reported results of the CI teams were compared to the outcomes of the formal CI evaluation for one of the accuracy measures (discordance in malaria cases between OPD register and monthly report; 8 months of data, 40 data points), and in a more limited fashion, the outcomes for clinically-relevant completeness (2–5 months following learning session 1 and 15 data points, limited by the records of the CI teams). Pearson’s correlation and the mean difference (per HF) between the CI team and evaluation team data were used to describe the comparability of the measured outcomes and to assess the potential bias of having the evaluation based solely on data collected by CI teams (a method often used to evaluate CI).

The cost-effectiveness evaluation considered training and other intervention costs, including transportation, per diem for the CI mentor and the CI teams during learning sessions, venue rentals, general miscellaneous training-related costs, and salaries of the CI implementer team. Costs incurred from the involvement of facility or MOH staff were not considered. Decision-tree analysis was used to calculate the cost-effectiveness of the intervention in terms of expenditure per additional malaria patient record that was complete. Inputs to the model are listed in Additional file 2: Annex 2. All costs were in US dollars based on the average Ugandan shilling 2016 exchange rate. Monte Carlo simulations were used to determine the point estimate and 95% confidence interval (95% CI) for the incremental cost-effectiveness ratio.

## Results

**Fig. 2:**
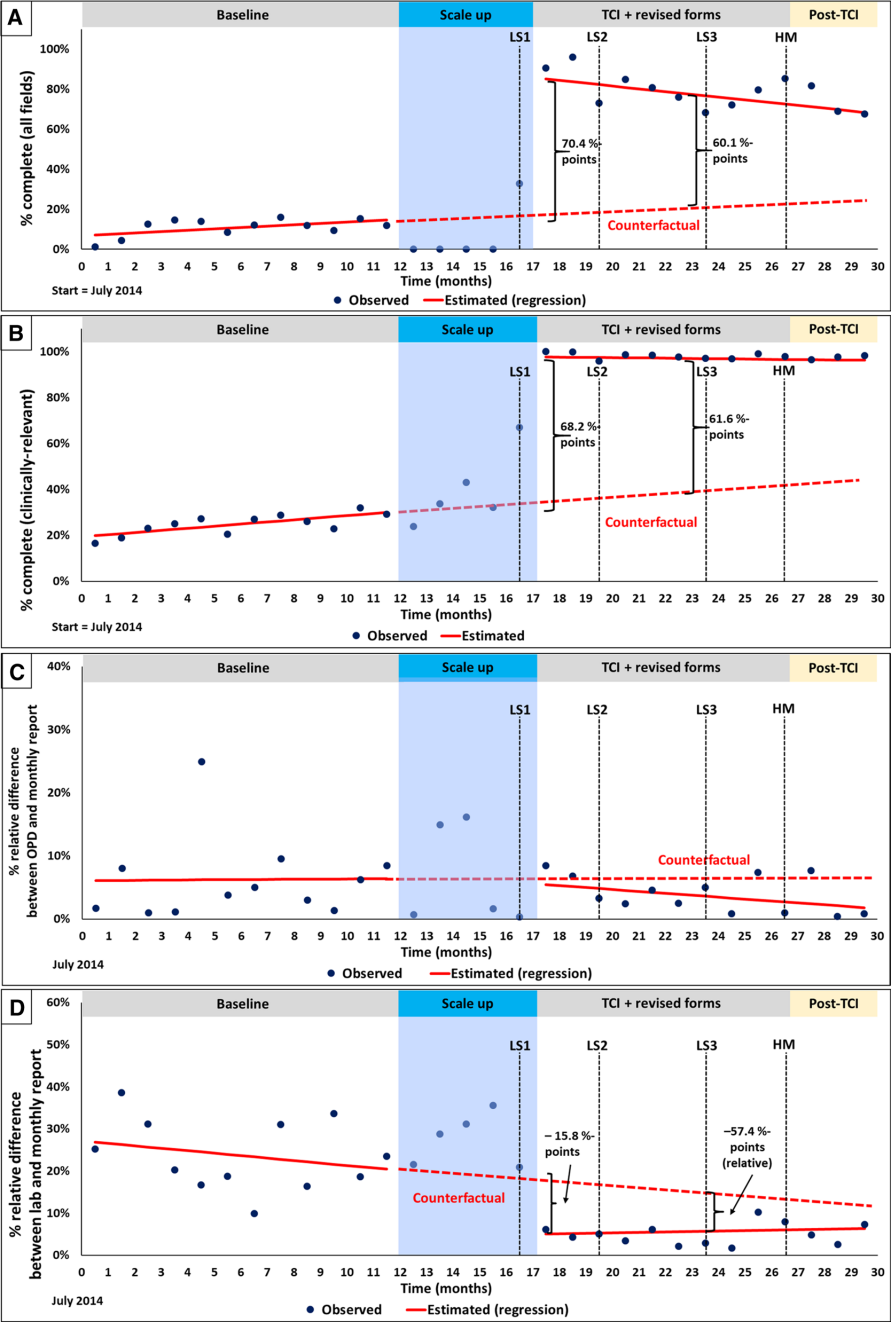
**A–D** Effect of TCI on **A** data completeness, all fields; **B** data completeness, clinically-relevant fields; **C** the relative difference (absolute value) between the OPD register and monthly reports for total malaria cases; **D** relative difference (absolute value) in test positivity rate based on the laboratory register data versus monthly reports. The graphs in **A**–**D** show three segments: (1) baseline, (2) scale-up of TCI and the revised forms (excluded from analysis), and (3) a period reflecting the effect of TCI and the revised forms followed by a short-term post-intervention sustainability assessment (post-TCI). The timing of the learning sessions (LS): LS1—focus on completeness; LS2—focus on accuracy through concordance of data sources; LS3—focus on data elements for calculation of standard malaria indicators); the harvest meeting (HM) completes the intervention period. Improvement in data quality is represented by positive effects sizes for completeness indicators (as the goal is 100% completeness) and negative effect sizes for accuracy indicators (as the goal is no discordance). *TCI* in-service training plus collaborative improvement, *LS* learning session, *HM* harvest meeting. Solid lines are based on the segmented regression model. The dashed line represents the counterfactual (an extension of the baseline trend into the follow-up period)

The evaluation team abstracted 156,707 entries from OPD and lab registers and aggregated monthly reports from July 2014 to December 2016. All-field patient register completeness was 15.0% at baseline with no significant change through the baseline period (0.7%-points per month; 95% CI: − 0.4, 1.7) (Table 2, column 1). A statistically significant immediate improvement (70.4%-points; 95% CI: 60.8, 80.0) and mid-point improvement (60.1%-points; 95% CI: 46.9, 73.2) was observed following the TCI intervention (Fig. 2A). Throughout the follow-up period, however, a significant negative change in slope was found (− 1.6%-points per month; 95% CI: − 2.8, − 0.3), which represents a decay of − 2.3%-points per month in effect size when accounting for the counterfactual slope, and which suggests a steady deterioration in TCI’s effect. By HF, heterogeneity in baseline trends and intervention was noted (Additional file 3: Annex 3). Unlike other HFs, health facility 2 (HF2) showed a lower level of improvement after TCI and was the only HF with a downward slope during follow-up. Also, HF3 had a higher baseline level of completeness with a considerable positive slope and reached a high level of completeness prior to TCI. To evaluate the effect of HF3 and its implausible counterfactual, a sensitivity analysis was performed (Table 1, Additional file 4: Annex 4) by excluding HC3 and found a greater immediate effect (82.5%-points vs. 70.4%-points) and non-significant deterioration in the slope over time during follow-up (− 0.9%-points, p = 0.17), in comparison to the original model.

**Table 2:** Effects of training plus collaborative improvement on data quality: results of segmented regression modelling

	Completeness Estimate (95% confidence interval) %-points		Accuracy Discordance between data sources† Estimate (95% confidence interval) %-points	
	All fields	All clinically-relevant fields	Malaria cases (OPD vs. report)	Test positivity rate (lab vs. report)
Baseline	15.0 (7.7, 22.2)***	30.5 (27.8, 33.1)***	6.7 (0.8, 12.6)*	20.1 (13.1, 27.2)***
Baseline slope (per month)	0.7 (− 0.4, 1.7)	0.9 (0.6, 1.3)***	0.1 (− 0.8, 0.9)	− 0.6 (− 1.6, 0.4)
Immediate change after TCI	70.4 (60.8, 80.0)***	68.2 (64.6, 71.7)***	− 0.9 (− 8.7, 6.9)†	− 15.8 (− 25.1, − 6.5)**†
Change in slope after TCI (per month)	− 1.6 (− 2.8, − 0.3)**	− 1.0 (− 1.5, − 0.6)***	− 0.3 (− 1.4, 0.7)†	0.7 (− 0.6, 1.9)†
TCI single effect (absolute difference at follow-up mid-point)	60.1 (46.9, 73.2)***	61.6 (56.6, 66.7)*	–	–
TCI single effect (relative difference at follow-up mid-point)	–	–	− 47.4 (− 177.0, 82.2)	− 57.4 (− 105.5, − 9.3)**

No significant autocorrelation was observed in the models

*TCI* in-service training plus collaborative improvement, *OPD* outpatient department register

^*^p < 0.05; **p < 0.01; ***p < 0.001. †Negative values for accuracy indicators are a sign of improvement

Baseline register completeness for selected clinically-relevant fields (age, sex, weight, diagnosis, and treatment) was 30.5% with a small but significant increase during the baseline period (0.9%-points per month; Table 2, column 2). Post-intervention, an immediate increase of 68.2%-points (95% CI: 64.6, 71.7) brought completeness to almost 100% across sites. This level of completeness was maintained throughout the follow-up period (Fig. 2B). The mid-point effect (61.6%-points; 95% CI: 56.6, 66.7) reflects TCI’s mean effect throughout the follow-up period. There was a significant negative change in slope (− 1.0%-point per month; 95% CI: − 1.5, − 0.6), but this did not reflect a decay in TCI’s effect. Rather, it was because the outcome trend flattened out at near 100% (and thus, the small positive baseline slope was essentially negated). Similar to the all-fields completeness outcome, trends varied by HF (mostly for the baseline period (see Additional file 3: Annex 3). Excluding HC3 had little effect on the results (Table 1, Additional file 4: Annex 4).

The baseline difference between the number of malaria cases recorded in OPD registers and the number of malaria cases submitted in monthly reports was relatively low at 6.7% with no significant change throughout baseline (slope: 0.1%-points per month), as shown in Table 2 and Fig. 2C. Following the intervention, no immediate change was observed (− 0.9%-points; 95% CI: − 8.7, 6.9) and the mid-point effect was not significant (− 47.4%-points; 95% CI: – 177.0, 82.2). Because the analyses were based on an absolute value of the discordance between the OPD registers and monthly reports, the dispersion of the discordance values and directionality in site-specific graphs (Additional file 5: Annex 5) was assessed, showing that, in most HFs, over- and under-reporting compared to OPD values had no observable pattern.

The baseline test positivity rate (proportion of all malaria tests done with a positive result; TPR) discordance between laboratory registers and monthly reports was 20.1% with no significant change throughout baseline (slope: − 0.6%-points per month), as shown in Table 2 and Fig. 2D. Discordance significantly decreased by 15.8%-points (95% CI: − 25.1, − 6.5) immediately post-TCI scale-up. The mid-point effect was a relative 57.4%-point improvement (95% CI: − 105.5, − 9.3) in accuracy. This estimate reflects a drop of 11.6%-points in the discrepancy between the two data sources, that is expressed as a 57.4%-point relative difference when compared to the counterfactual. The non-significant slope during the follow-up period indicates that achievements were sustained. Similar to the malaria cases accuracy indicator, the graphs in Additional file 5: Annex 5 show different patterns of discordance across HF over time.

Timeliness, which was initially included as a study outcome, was dropped from the analysis because it was already high (> 95%) at baseline and had little room to improve.

The agreement in results between facility-based CI teams and study evaluation teams was moderate: Pearson’s correlation for the accuracy outcome (discordance in malaria cases between OPD register and monthly report) was 0.43, ranging from − 0.06 for HF5 (level II) to 0.99 for HF1 (level IV); and 0.68 for the clinically-relevant completeness outcome. Mean differences in the accuracy by CI teams compared to the evaluation team data (i.e., CI team result minus evaluation team result) were − 2%-points for all sites, and ranged from −15%-points to − 3%-points for higher level HFs (HC IIIs and HC IVs) and 4–6%-points for lower level HFs (level II). Negative values indicate that CI teams found greater reductions in discrepancies between the data sources, and thus a greater improvement in accuracy of data, compared to the evaluation team’s results (results and scatterplots reported in Additional file 6: Annex 6).

The total calculated cost of the TCI intervention implemented at the five pilot HFs was 51,399 USD. Cost-effectiveness results estimate a cost of 3.53 USD (95% CI: 3.03, 4.15 USD) per malaria patient record with complete clinically-relevant data recorded.

## Discussion

Surveillance data are essential for malaria control, but data quality is often poor. CI is a promising quality improvement intervention, but it has not been rigorously evaluated as a method to improve surveillance data quality. To evaluate the impact of brief in-service training plus the CI methodology on quality of malaria surveillance data, a pilot study was conducted in 5 HF in Kayunga, Uganda.

Overall, the study found that in-service training and CI resulted in large improvements in data completeness, with limited effect on accuracy. Specifically, the estimates of the immediate and mid-point effect of the two completeness indicators (all-field completeness: 70.4% points and 60.1%-points, respectively; clinically-relevant completeness: 68.2%-points and 61.6%-points, respectively) align with the evidence of efficacy of training with CI from three collaboratives from Niger [51]. Although the Niger collaboratives addressed maternal and newborn care rather than malaria surveillance, the indicators addressing adherence to care standards could be relevant for comparison with the data quality standards in this study. Across the Niger collaboratives, adherence to standards was improved for the following aspects of care: measuring the newborns’ temperature (by 60.9% and 96.5% in two collaboratives); vaginal deliveries according to active management in third stage of labour (by 89.6% and 91.4% in two collaboratives); essential newborn care (by 71.0% and 85.7% in two collaboratives); and pre-eclampsia and eclampsia by 35.3% in one collaborative. Overall, the Niger collaboratives documented higher levels of improvement, though these studies were classified as high risk of bias by the review [51]. In this study, data accuracy was high at baseline and thus, there was less room for improvement, which is encouraging. A significant improvement in data accuracy (as measured by the reduction in discrepancy between reporting in the laboratory registers and monthly reports) was found only for test positivity rate (relative improvement of 54.7%-points). In addition to the relatively high baseline, other factors that may have led to a less pronounced effect of CI on accuracy include: more complex mechanisms behind such improvement that needed collaboration and harmonization across different HF departments (e.g., outpatient, laboratory, pharmacy), as well as the shorter period of time the HF teams spent working on accuracy indicators, with the focus shifting to accuracy in Phase II of the intervention period.

A considerable heterogeneity in the intervention effects in site-specific analyses was observed. Four out of five HFs achieved high levels of completeness shortly after TCI was scaled up. But, at HF2, the outlier, little impact on all-field completeness and clinically-relevant completeness was observed. In the aggregate analysis, the poor performance of HF2 was the driving force behind the deterioration of the TCI effect for all-field completeness over time. HF3 achieved high levels of completeness during the baseline period, even before the intervention, for unclear reasons. A sensitivity analysis with and without HF3 was conducted that showed a greater immediate effect and non-significant deterioration in the slope over time during follow-up. Great variability in trends was also observed for the accuracy indicators before and after the intervention; harmonization of improvement trends was more pronounced for the TPR accuracy indicator. The study results should be interpreted with the understanding of this underlying heterogeneity by HF.

The correlation between data collected by the CI team and through the evaluation was moderate, with considerable variation across different HFs. The small mean difference (− 2%-points) for all sites was driven by the variation in the magnitude and directionality across sites and, therefore, does not indicate a good agreement between self-reported and evaluation-derived data. Because of differences in data collection (i.e., length of baseline and tracking periods, and differences in outcomes measures), the evaluation could not apply the same methods to CI teams’ data to estimate effect size. However, based on the correlation analyses, the differences suggest that the effect sizes calculated from the CI teams’ data would have been larger than those based on the evaluation data. By HF, the study results suggest that in higher level HFs (HC IIIs and HC IVs), CI teams may have overestimated the impact of TCI on data accuracy, whereas the opposite was true for lower level HFs.

The cost of TCI per additional case with complete recording of clinically-relevant data was $3.53 USD, which is similar to the estimated cost per additional malaria case treated appropriately in Uganda ($3 to $13 USD/case) [52] and the cost of improving community-based access to malaria care ($1.42 to $4.46 USD) [53]. If taken to scale, total TCI costs would increase, but because a coach can cover a larger number of HFs in non-research settings, per-patient costs would likely decrease. The opportunity costs of the health workers (HF employees) would increase the cost of the overall intervention by 10%, leading to a cost of $3.88 per additional case with complete recording of clinically-relevant data. However, if the goal was 100% coverage, there would be a point of diminishing marginal returns as covering the most remote or difficult HFs would be more costly and improve coverage only slightly. Finally, there could be a reduction in both cost and effect sizes, if the intervention were carried out by the MOH in conditions of insufficient human or financial resources.

### Strengths and limitations

An independent assessment of the intervention was conducted, focusing on the completeness of register data (a key internal measure of quality for HFs) and how well the monthly reports (the only source of HF information available to the MOH for calculating malaria indicators) reflect the data collected by HFs. This study was more rigorous that previous CI studies because it included an independent evaluation, used a longer baseline period, and used an objective data source for the evaluation (information from registers and monthly reports abstracted by a trained data collection team). Moreover, the study included both quantitative and qualitative evaluations [48, 50] to explore whether (or to what extent) CI worked, and how it worked. The TCI intervention followed the CI methodology, aside from scale: only 5 HFs were included in this proof-of-concept pilot, compared to the recommended network of 20–100 HFs for a well-established implementation package. The decision to combine training with CI was based on preliminary results of a systematic review that showed larger effect sizes from this combination compared to CI alone, albeit based on studies that generally had a high risk of bias [32].

The study had several limitations. First, the sample size was small. While three levels of HFs were included in the study (II, III, and IV), the results might not be generalizable to the reporting practices and effect of the CI approach of the entire healthcare system in Uganda. However, the included HFs are fairly typical for Uganda in terms of their recording and reporting practices. Conducting the study in a single district (Kayunga) also limits the geographical representativeness of the study. However, the intent of this study was to be an in-depth exploration, both quantitative and qualitative, of the inner workings of the CI method for improvement of the surveillance data quality, and this purpose was best realized through a small pilot study due to the large efforts required to conduct the qualitative work.

No data were collected except those directly related to the intervention’s effect on routine malaria data quality. However, it is possible that other aspects of performance of the health workers in health service delivery may have changed. On one hand, it is possible that the improvement methods implemented during this intervention may have been used independently by health workers to improve other aspects of their performance. For example, clinicians may have used the same methods to increase the proportion of non-malaria patient records with complete clinically relevant data. Conversely, it is possible that other aspects of data quality may have worsened because clinicians were focused on malaria data, to the detriment of other work. However, because only malaria-specific data were collected, the impact beyond the primary focus of the intervention could not be assessed. The qualitative component of the study evaluated the unintended consequences of the intervention (e.g., effects of changes to patient flow to better capture data on the patient waiting time) from multiple angles, as reported by Hutchinson et al*.* [48, 50]. Future studies should collect data on a wider spectrum of quality indicators to examine spill-over effects, which could be positive or negative.

The brief time between the introduction of the revised forms by the MOH and the start of the study intervention precluded meaningful analysis of the scale-up of the revised forms in the time series design and segmented regression analysis. Therefore, it was not possible to fully evaluate the effect of introducing the revised forms on HF data quality. Similarly, because the study intervention included the in-service training plus CI, the study was not designed to assess the effects of the individual components of the intervention separately, although a recent comprehensive systematic review sheds some light on the effects of these components [32].

## Conclusions

This rigorously designed pilot study found that the TCI intervention was associated with large improvements in completeness, but not accuracy, of routinely collected malaria surveillance data. Future evaluations of TCI should ideally employ a cluster-randomized trial design with a larger sample size and longer follow-up period. It would be also recommended that the content of TCI be expanded to testing and treatment practices for malaria, as well as to other diseases. Sustainability evaluations and larger studies are needed before recommending wide-scale implementation of the CI intervention.

## Supplementary Information

**Additional file 1:** **Annex 1**. The change package resulting from the TCI intervention.

**Additional file 2:** **Annex 2**. Inputs for cost-effectiveness decision tree.

**Additional file 3:Annex 3**. Health-facility specific graphs.

**Additional file 4:** **Annex 4**. Sensitivity analyses and models by study site for the completeness indicators.

**Additional file 5**: **Annex 5**. Directionality and magnitude of dispersion of the accuracy indicators by study site.

**Additional file 6**: **Annex 6**. Correlation between the accuracy (discordance in malaria cases between OPD register and monthly report) and clinically-relevant completeness data collected through independent evaluation and data reported by the HF-based CI teams.

## Data Availability

The datasets used and/or analysed during the current study are available from the corresponding author on reasonable request.

## Abbreviations

HMIS: Health Management Information Systems
CI: Collaborative improvement
PDSA: Plan-do-study-act
WHO: World Health Organization
LMIC: Low- and middle-income country
HC: Health Centre
OPD: Out-patient department
FGD: Focus group discussion
USD: United States dollars
CDC: Centers for Disease Control and Prevention
