# Supplementary material for: Effectiveness of in-service training plus the collaborative improvement strategy on the quality of routine malaria surveillance data: results of a pilot study in Kayunga District, Uganda
Source: Malar J. 2021 Jun 29;20:290. doi: 10.1186/s12936-021-03822-y (PMC8243434; doi:10.1186/s12936-021-03822-y)
Supplement: Supplementary file 1 — Additional file 1: Annex 1. The change package resulting from the TCI intervention. [file 12936_2021_3822_MOESM1_ESM.docx]

**Effectiveness of in-service training plus the collaborative improvement strategy on the quality of routine malaria surveillance data: results of a pilot study in Kayunga District, Uganda**

## Annex 1. The change package resulting from the TCI intervention

A central element of a learning collaborative, the change package is a catalogue of tried strategies, change concepts, and actionable items that document the effective changes that led to improvements made in the course of a collaborative. This [change package](https://www.nnoha.org/nnoha-content/uploads/2017/06/Dashboard-Collaborative-Pilot-CHANGE-PACKAGE-June-2017-revised-5-30-17.pdf) is a compilation of the changes and strategies that HF-based CI teams in Uganda have tested and found worked best to improve the completeness and accuracy of the malaria surveillance and can be used by the individual health facilities or the Ministry of Health at scale to improve the malaria surveillance.

Abbreviations:

ACT Artemisinin-based combination therapy

ANC Antenatal Clinic

CME Continuous Medical Education

IPD Inpatient Department

mRDT Malaria Rapid Diagnostic Test

OPD Outpatient Department

VHT Village Health Team

| **Tested change idea** | **Perceived effectiveness** | | **Relative importance** | **Simplicity/ scalability** | **Minimal resources needed** | **Total rating** |
| --- | --- | --- | --- | --- | --- | --- |
| **Completeness of Outpatient (OPD) register** |  | |  |  |  |  |
| - Assign OPD numbers before laboratory investigations are done (start of visit) | 5.0 | | 5.0 | 5.0 | 5.0 | 20.0 |
| - Assign a focal person to monitor completeness of OPD register daily and fill the gaps | 5.0 | | 5.0 | 5.0 | 5.0 | 20.0 |
| - Establish a registration / prescription area | 5.0 | | 5.0 | 4.0 | 4.0 | 18.0 |
| - Develop a checklist of commonly missed parameters | 5.0 | | 5.0 | 3.0 | 5.0 | 18.0 |
| - Assign a person to take measurements and record in the OPD register | 3.0 | | 4.0 | 5.0 | 5.0 | 17.0 |
| - Assign a second focal person to monitor completeness of the OPD register | 3.0 | | 4.5 | 3.5 | 5.0 | 16.0 |
| - Orient staff to fill the OPD register through CMEs | 1.0 | | 4.0 | 5.0 | 5.0 | 15.0 |
| - Assign someone to pick patients books from the laboratory, record in the OPD register and take the book to the clinician | | 2.0 | 5.0 | 3.0 | 5.0 | 15.0 |
| - Mentor village health team (VHT) volunteers to complete the OPD registers | 2.0 | | 5.0 | 3.0 | 3.0 | 13.0 |
| - Redesign OPD patient flow | 3.0 | | 2.0 | 2.0 | 4.0 | 11.0 |
| - Assign a person to transfer patient information from patients’ books to the OPD register before leaving the facility (end of visit) | 2.0 | | 2.0 | 2.0 | 3.0 | 9.0 |
| **Accuracy (concordance between data sources)** |  | |  |  |  |  |
| ***Concordance between OPD register and monthly report*** |  | |  |  |  |  |
| - Crosscheck daily summaries with monthly totals in the monthly reports | 5.0 | | 5.0 | 5.0 | 5.0 | 20.0 |
| - Introduce daily tally book to generate daily summaries of patients diagnosed with malaria | 4.6 | | 4.6 | 4.2 | 4.2 | 17.6 |
| - Second person recounts number of malaria cases in the OPD register | 4.5 | | 4.5 | 4.0 | 4.0 | 17.0 |
| - Check daily to ensure the word malaria for diagnosis is not abbreviated | 3.0 | | 5.0 | 4.0 | 4.0 | 17.0 |
| - Generate weekly summaries of all patients diagnosed with malaria | 3.0 | | 3.0 | 4.0 | 4.0 | 14.0 |
| ***Concordance: number of malaria tests between laboratory and OPD registers*** |  | |  |  |  |  |
| - VHT/ health worker collects patient books from OPD and laboratory to clinicians | 5.0 | | 5.0 | 5.0 | 5.0 | 20.0 |
| - Redesign OPD patient flow | 4.0 | | 4.0 | 5.0 | 5.0 | 18.0 |
| - Assign compulsory OPD numbers before laboratory investigations are done | 4.0 | | 5.0 | 3.0 | 5.0 | 17.0 |
| - Involve VHTs and facility linkage facilitators in recording results in the OPD register | 3.0 | | 3.0 | 4.0 | 4.0 | 14.0 |
| **Tested change idea** | **Perceived effectiveness** | | **Relative importance** | **Simplicity/ scalability** | **Minimal resources needed** | **Total rating** |
| ***Concordance: patients with positive malaria test results between lab and OPD registers*** |  | |  |  |  |  |
| - Tick the patient book to indicate patient details have been entered in the OPD register | 5.0 | | 5.0 | 5.0 | 5.0 | 20.0 |
| - VHT/ health worker collects patient books from OPD and laboratory to clinicians | 5.0 | | 5.0 | 5.0 | 5.0 | 20.0 |
| - Orient new staff on how to complete the OPD register | 4.0 | | 4.0 | 5.0 | 5.0 | 18.0 |
| - Spot check the OPD register to ensure all patients have malaria test results recorded | 4.0 | | 4.0 | 3.5 | 4.5 | 16.0 |
| - Prescribe patient medication only within the clinician’s room | 4.0 | | 4.0 | 3.0 | 4.0 | 15.0 |
| - Change from ticking in designated spaces to writing POS and NEG for malaria results | 3.5 | | 4.0 | 3.0 | 3.5 | 14.0 |
| - Shift staff who are unwilling to record well in the OPD register to another department | 3.0 | | 3.0 | 2.0 | 2.0 | 10.0 |
| ***Concordance: pharmacy and OPD records*** |  | |  |  |  |  |
| - Crosscheck dispensing log and OPD register every morning by the first arriving staff | 5.0 | | 5.0 | 5.0 | 5.0 | 20.0 |
| - Record 00 in the dispensing log whenever there are stockouts of ACTs | 5.0 | | 5.0 | 4.7 | 4.7 | 19.3 |
| - Report early for duty to check for gaps | 3.0 | | 5.0 | 4.0 | 5.0 | 17.0 |
| - Tally number of ACTs dispensed to malaria positive patients in the OPD register daily | 4.0 | | 5.0 | 3.0 | 5.0 | 17.0 |
| - Assign one person to record at a given period | 4.0 | | 4.0 | 5.0 | 5.0 | 17.0 |
| - Mentor staff on filling the dispensing log | 3.0 | | 3.0 | 3.0 | 3.0 | 12.0 |
| - Improvise the first column in the dispensing log for ACTs (recording 00) | 1.0 | | 1.0 | 1.0 | 1.0 | 4.0 |
| **Malaria case management changes (as a product of records improvement)** |  | |  |  |  |  |
| ***Test and treat: patients with suspected malaria tested*** |  | |  |  |  |  |
| - Assign a triage nurse or a VHT to send all patients with fever to the laboratory | 5.0 | | 5.0 | 5.0 | 5.0 | 20.0 |
| - Display the test and treat policy | 5.0 | | 5.0 | 5.0 | 5.0 | 20.0 |
| - Create testing points like antenatal clinic (ANC), OPD and IPD on weekends / nights | 5.0 | | 5.0 | 5.0 | 5.0 | 20.0 |
| - Conduct CME on test and treat policy | 4.5 | | 5.0 | 4.5 | 5.0 | 19.0 |
| - All malaria suspects get a laboratory request form filled by a clinician | 4.0 | | 4.0 | 5.0 | 5.0 | 18.0 |
| - Manage malaria rapid diagnostic test (mRDT) stock daily | 4.0 | | 4.0 | 4.0 | 4.0 | 16.0 |
| ***Test and treat: patients treated for malaria have a confirmed malaria test*** |  | |  |  |  |  |
| - Tick to indicate the patient is treated with a positive malaria test | 5.0 | | 5.0 | 5.0 | 5.0 | 20.0 |
| - Educate patients about test and treat policy | 5.0 | | 5.0 | 5.0 | 5.0 | 20.0 |
| - Conduct CME on test and treat policy | 5.0 | | 5.0 | 4.7 | 5.0 | 19.7 |
| - Avail mRDTs to ANC | 5.0 | | 4.0 | 3.0 | 5.0 | 17.0 |

Pearson correlation = 0.68

Mean difference (mCI – mEval) = 10%-points (SD: 23%-points)***

***positive values are less conservative
