# Supplementary material for: Effectiveness of in-service training plus the collaborative improvement strategy on the quality of routine malaria surveillance data: results of a pilot study in Kayunga District, Uganda
Source: Malar J. 2021 Jun 29;20:290. doi: 10.1186/s12936-021-03822-y (PMC8243434; doi:10.1186/s12936-021-03822-y)
Supplement: Supplementary file 2 — Additional file 2: Annex 2. Inputs for cost-effectiveness decision tree. [file 12936_2021_3822_MOESM2_ESM.docx]

**Effectiveness of in-service training plus the collaborative improvement strategy on the quality of routine malaria surveillance data: results of a pilot study in Kayunga District, Uganda**

# Annex 2. Inputs for cost-effectiveness decision tree

| **Model input** | **Estimate** | **Distribution** |
| --- | --- | --- |
| Pre-intervention data completeness | 36.6% | Binomial |
| Post-intervention data completeness | 98.1% | Binomial |
|  |  |  |
| **Cost** | **$** | |
| Cost of coaching | 2,503 | |
| Cost of Kampala learning sessions (2) | 13,324 | |
| Cost of Kayunga learning session (1) | 2,610 | |
| Cost of in-service training (1) | 2,610 | |
| Miscellaneous costs | 2,881 | |
| Improvement experts salaries | 27,471 | |
| **Total** | **51,399** | |
|  | | |
| **Denominator (n)** | **n** | |
| Malaria patient population | 25,759 | |
