# Supplementary material for: Effectiveness of in-service training plus the collaborative improvement strategy on the quality of routine malaria surveillance data: results of a pilot study in Kayunga District, Uganda
Source: Malar J. 2021 Jun 29;20:290. doi: 10.1186/s12936-021-03822-y (PMC8243434; doi:10.1186/s12936-021-03822-y)

**Effectiveness of in-service training plus the collaborative improvement strategy on the quality of routine malaria surveillance data: results of a pilot study in Kayunga District, Uganda**

# Annex 3. Health-facility specific graphs

Abbreviations: TCI – training plus collaborative improvement; HF – health facility


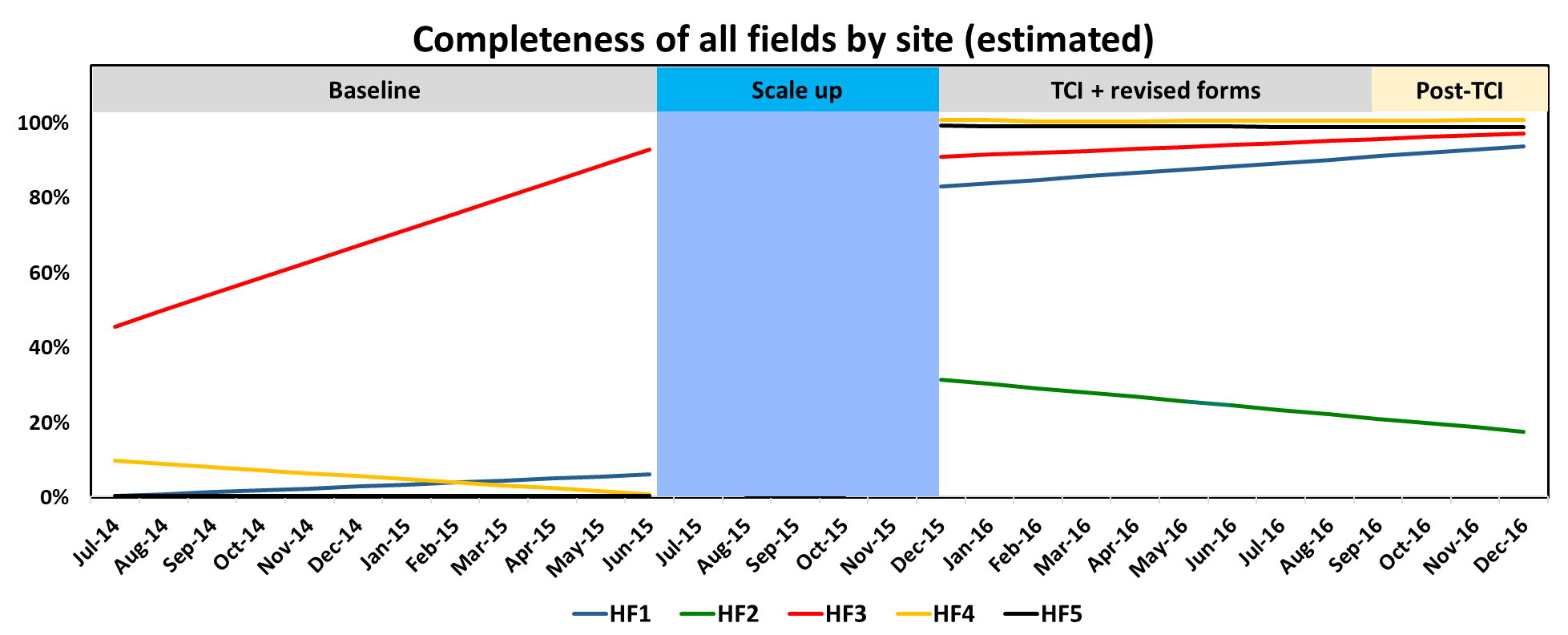


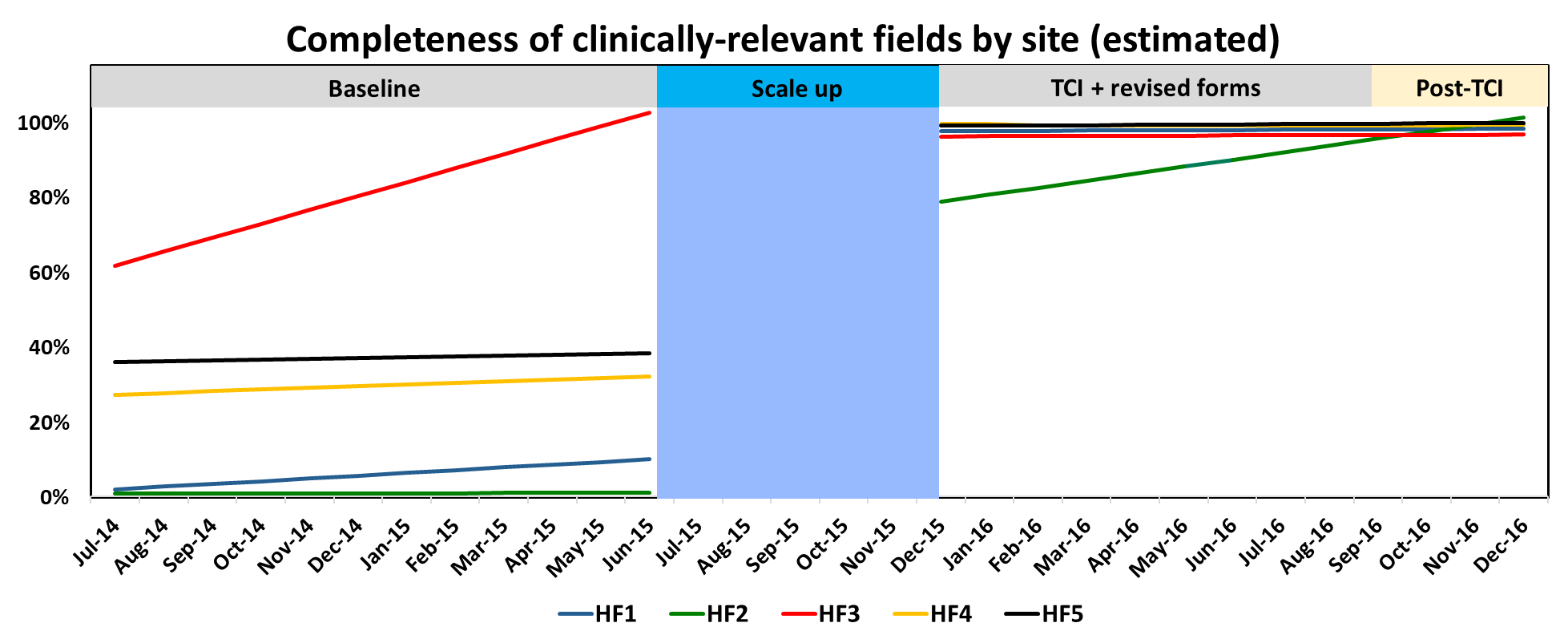


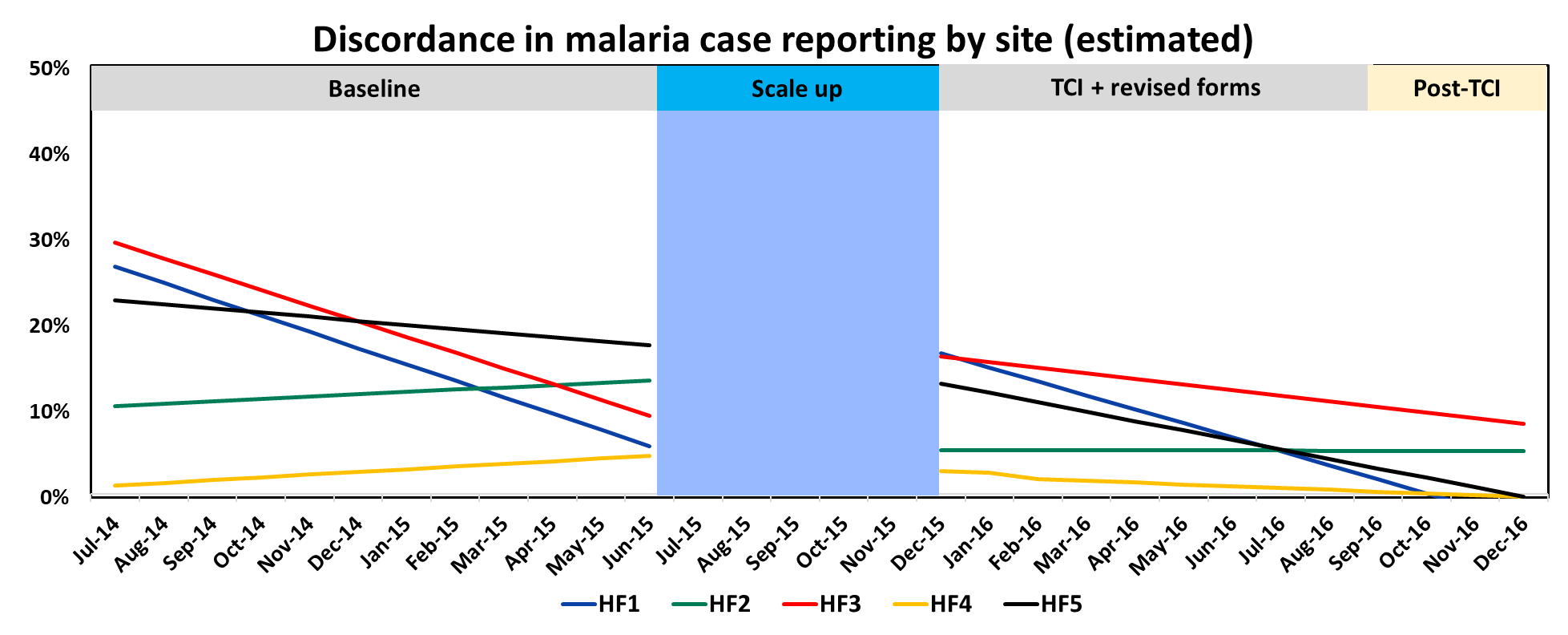


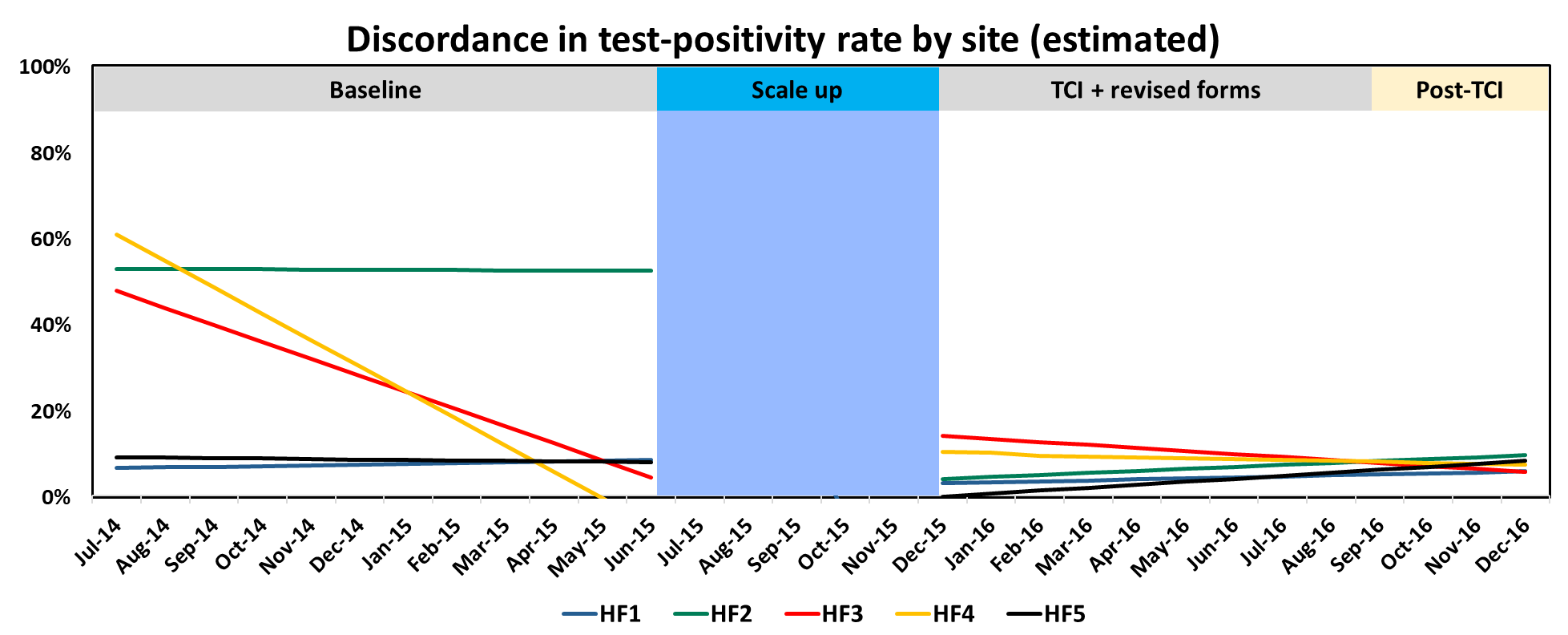

Supplement: Supplementary file 3 — Additional file 3:Annex 3. Health-facility specific graphs. [file 12936_2021_3822_MOESM3_ESM.docx]
