# Supplementary material for: Effectiveness of in-service training plus the collaborative improvement strategy on the quality of routine malaria surveillance data: results of a pilot study in Kayunga District, Uganda
Source: Malar J. 2021 Jun 29;20:290. doi: 10.1186/s12936-021-03822-y (PMC8243434; doi:10.1186/s12936-021-03822-y)
Supplement: Supplementary file 4 — Additional file 4: Annex 4. Sensitivity analyses and models by study site for the completeness indicators. [file 12936_2021_3822_MOESM4_ESM.docx]

**Effectiveness of in-service training plus the collaborative improvement strategy on the quality of routine malaria surveillance data: results of a pilot study in Kayunga District, Uganda**

## Annex 4. Sensitivity analyses and models by site for the completeness indicators

Table 1. Results of the segmented regression analyses for the completeness of all fields and completeness of clinically-relevant fields: models with all HFs included and models with HF3 excluded due to the implausible counterfactual (sensitivity analyses)

|  | **Completeness, all fields Estimate (95% confidence interval)**  **%-points** | | **Completeness, clinically-relevant Estimate (95% confidence interval)**  **%-points** | |
| --- | --- | --- | --- | --- |
|  | **All HF** | **Excluding HF3** | **All HF** | **Excluding HF3** |
| **Baseline** | 15.0 (7.7, 22.2)* | 0.4 (-8.0, 8.7) | 30.5 (27.8, 33.1)* | 18.1 (15.5, 20.6)* |
| **Baseline slope (per month)** | 0.7 (-0.4, 1.7) | -0.2 (-1.4, 1.0) | 0.9 (0.6, 1.3)* | 0.4 (0.0, 0.7) |
| **Immediate change after TCI** | 70.4 (60.8, 80.0)* | 82.5 (70.7, 94.2)* | 68.2 (64.6, 71.7)* | 80.1 (76.4, 83.7)* |
| **Change in slope after TCI (per month)** | -1.6 (-2.8, -0.3)* | -0.9 (-2.1, 0.3) | -1.0 (-1.5, -0.6)* | -0.4 (-0.7, 0.0) |
|  |  |  |  |  |

*p<0.05; TCI – training plus collaborative improvement

Figure 1A-B. Observed and estimated values of the all-fields completeness indicator. A) Complete model (all HF); B) HF3 excluded.


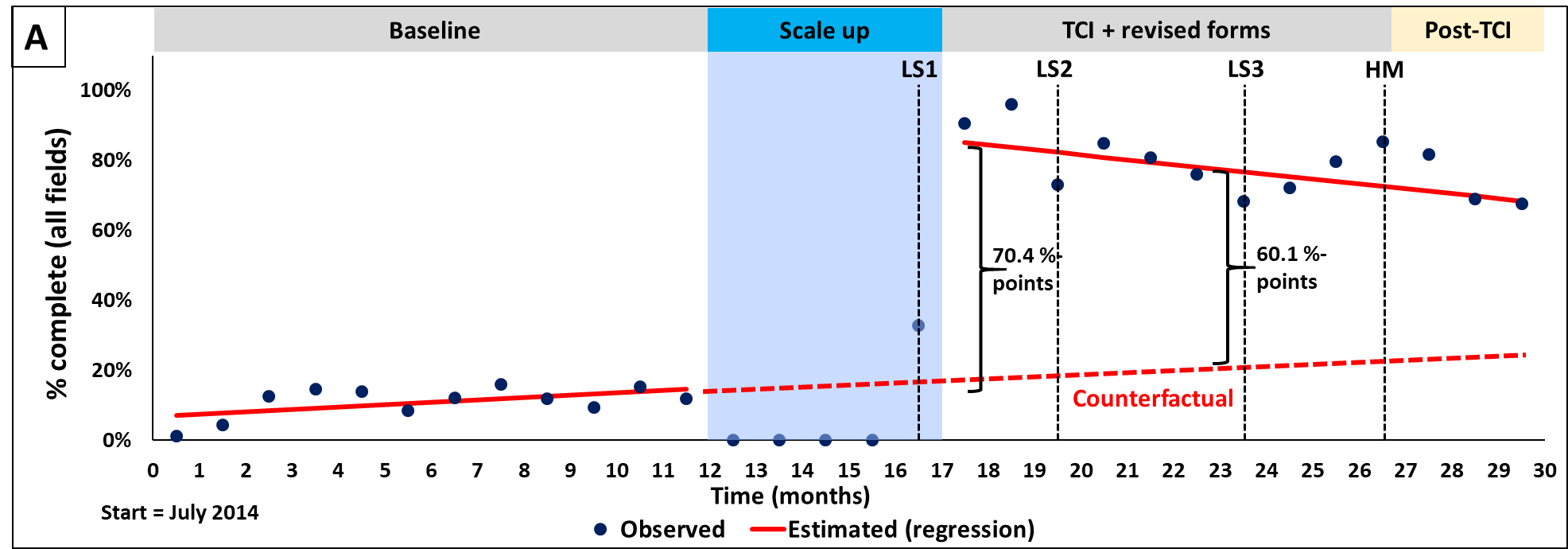


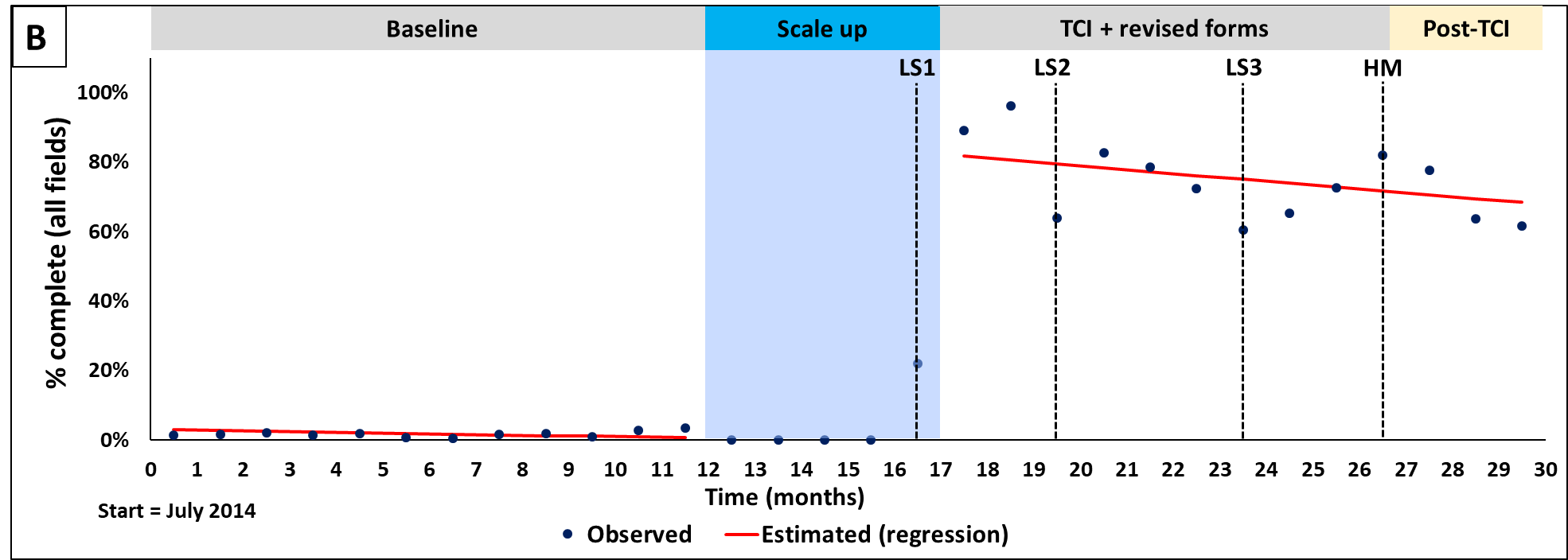


Figure 2A-B. Observed and estimated values of the clinically-relevant field completeness: A) Complete model (all HF); B) HF3 excluded.


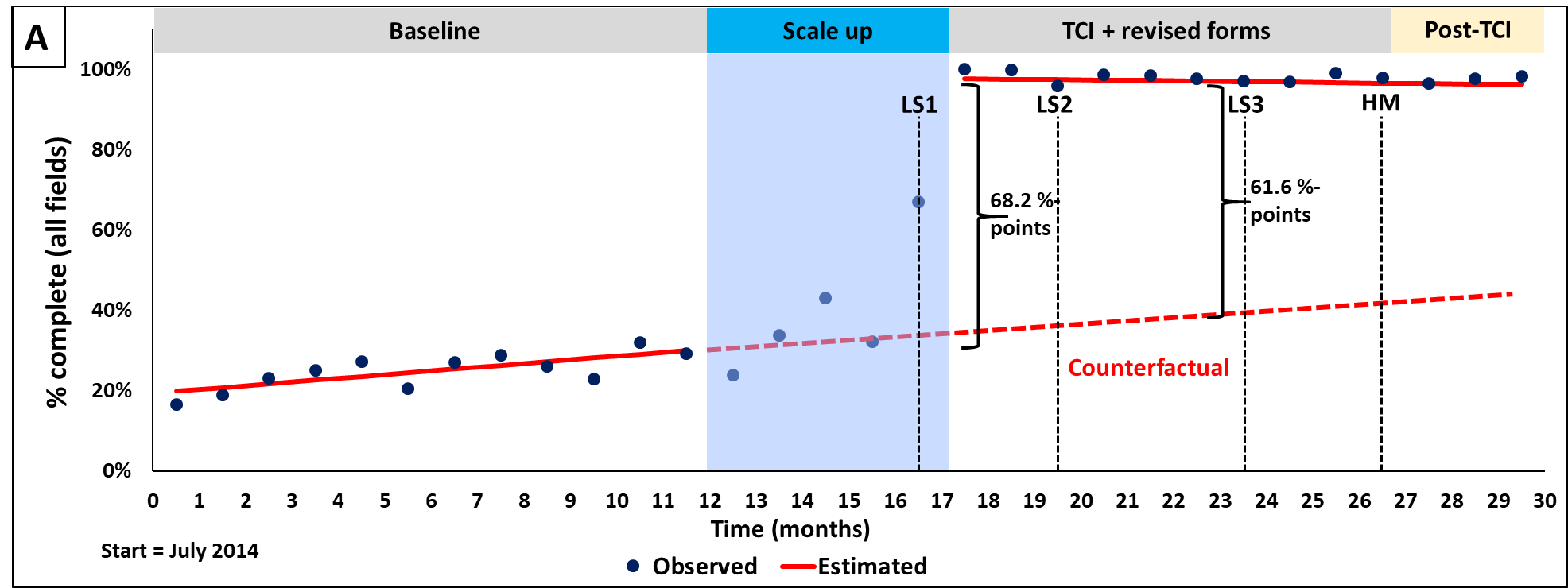


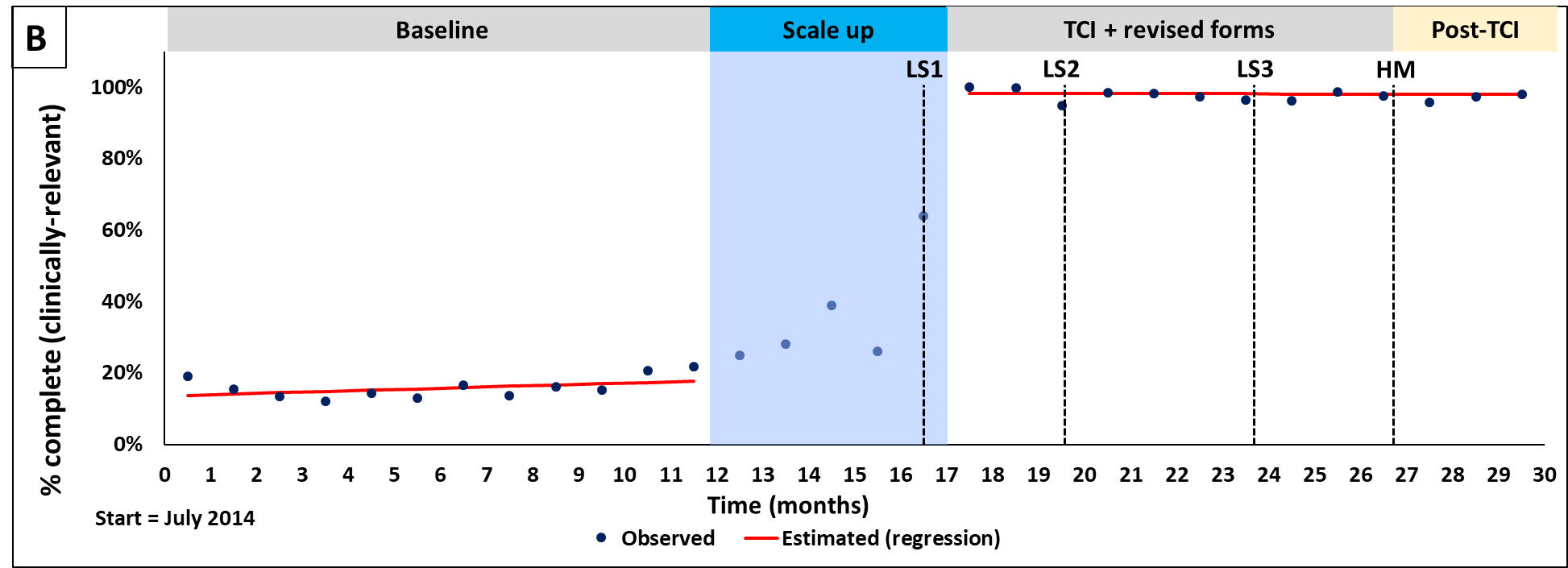


Table 2. Site-specific results of the segmented regression analyses for all-field completeness

|  | **HF1**  **% (95%CI)** | **HF2**  **% (95%CI)** | **HF3**  **% (95%CI)** | **HF4**  **% (95%CI)** | **HF5**  **% (95%CI)** |
| --- | --- | --- | --- | --- | --- |
| **Baseline** | 3.7 (-5.7, 13.0) | -0.8 (-10.3, 8.7) | 82.8 (53.1, 112.4)* | 0.2 (-3.0, 3.5) | 0.2 (-1.3, 1.7) |
| **Baseline slope (per month)** | 0.1 (-1.2, 1.5) | -0.1 (-1.5, 1.2) | 4.2 (0.2, 8.1)* | -0.8 (-1.2, -0.3)* | 0.0 (-0.2, 0.3) |
| **Immediate change after TCI** | 76.6 (63.4, 89.8)* | 32.6 (19.3, 46.0)* | 20.2 (-9.9, 50.2) | 99.8 (95.2, 104.3)* | 98.8 (96.7, 101.0)* |
| **Change in slope after TCI (per month)** | 0.8 (-0.6, 2.2) | -0.7 (-2.1, 0.7) | -3.5(-8.2, 1.3) | 0.6 (0.1, 1.1)* | -0.1 (-0.3, 0.1) |
|  |  |  |  |  |  |

Table 3. Site-specific results of the segmented regression analyses for clinically-relevant field completeness

|  | **HF1**  **% (95%CI)** | **HF2**  **% (95%CI)** | **HF3**  **% (95%CI)** | **HF4**  **% (95%CI)** | **HF5**  **% (95%CI)** |
| --- | --- | --- | --- | --- | --- |
| **Baseline** | 10.0 (5.3, 14.7)* | 2.7 (-9.4, 14.8) | 93.9 (77.4, 110.5)* | 32.1 (28.4, 35.8)* | 38.3 (33.3, 43.3)* |
| **Baseline slope (per month)** | 0.7 (0.0, 1.4) | 0.3 (-1.4, 2.1) | 4.2 (1.8, 6.5)* | 0.4 (-0.1, 1.0) | 0.2 (-0.5, 0.9) |
| **Immediate change after TCI** | 87.6 (81.0, 94.2)* | 75.1 (58.1, 92.2)* | -1.9 (-25.2, 21.4) | 67.4 (62.2, 72.6)* | 60.6 (53.6, 67.6)* |
| **Change in slope after TCI (per month)** | -0.5 (-1.2, 0.2) | 1.0 (-0.8, 2.8) | -2.7 (-5.1, -0.3)* | -0.3 (-0.8, 0.2) | -0.1 (-0.9, 0.6) |
|  |  |  |  |  |  |
