# Supplementary material for: Effectiveness of in-service training plus the collaborative improvement strategy on the quality of routine malaria surveillance data: results of a pilot study in Kayunga District, Uganda
Source: Malar J. 2021 Jun 29;20:290. doi: 10.1186/s12936-021-03822-y (PMC8243434; doi:10.1186/s12936-021-03822-y)
Supplement: Supplementary file 5 — Additional file 5: Annex 5. Directionality and magnitude of dispersion of the accuracy indicators by study site. [file 12936_2021_3822_MOESM5_ESM.docx]

**Effectiveness of in-service training plus the collaborative improvement strategy on the quality of routine malaria surveillance data: results of a pilot study in Kayunga District, Uganda**

## Annex 5. Directionality and magnitude of dispersion of the accuracy indicators by study site

**Accuracy indicator #1: Discordance in malaria cases reported in the OPD register vs. monthly report (separate graphs showing the relative differences and the actual numbers of malaria patients those differences were derived from)**

Abbreviations: OPD – outpatient department; TCI – training and collaborative improvement

**TCI scale up**

**TCI scale up**

**TCI scale up**

**TCI scale up**

**TCI scale up**

**TCI scale up**

**TCI scale up**

**TCI scale up**

**TCI scale up**

**TCI scale up**

**Accuracy indicator #2: Discordance in test positivity rate (TPR) calculated from the lab register vs. monthly report (separate graphs showing the relative differences and the actual TPRs those differences were derived from)**

Abbreviations: TPR – test-positivity rate; TCI – training and collaborative improvement

**TCI scale up**

**TCI scale up**

**TCI scale up**

**TCI scale up**

**TCI scale up**

**TCI scale up**

**TCI scale up**

**TCI scale up**

**TCI scale up**

**TCI scale up**
