# Supplementary material for: Effectiveness of in-service training plus the collaborative improvement strategy on the quality of routine malaria surveillance data: results of a pilot study in Kayunga District, Uganda
Source: Malar J. 2021 Jun 29;20:290. doi: 10.1186/s12936-021-03822-y (PMC8243434; doi:10.1186/s12936-021-03822-y)
Supplement: Supplementary file 6 — Additional file 6: Annex 6. Correlation between the accuracy (discordance in malaria cases between OPD register and monthly report) and clinically-relevant completeness data collected through independent evaluation and data reported by the HF-based CI teams. [file 12936_2021_3822_MOESM6_ESM.docx]

**Effectiveness of in-service training plus the collaborative improvement strategy on the quality of routine malaria surveillance data: results of a pilot study in Kayunga District, Uganda**

**Annex 6. Correlation between the accuracy (discordance in malaria cases between OPD register and monthly report) and clinically-relevant completeness data collected through independent evaluation and data reported by the HF-based CI teams.**

Abbreviations: mCI = mean value of an indicator based on the CI team data; mEval = mean value of an indicator based on the evaluation data; SD = standard deviation

Pearson correlation = 0.43

Mean difference (mCI – mEval) = –2%-points (SD: 17%-points)

*negative values are less conservative

Pearson correlation = 0.99

Mean difference (mCI – mEval) = –3%-points (SD: 2%-points)*

*negative values are less conservative

Pearson correlation = 0.49

Mean difference (mCI – mEval) = –15%-points (SD: 29%-points)*

*negative values are less conservative

Pearson correlation = 0.63

Mean difference (mCI – mEval) = –3%-points (SD: 3%-points)*

*negative values are less conservative

Pearson correlation = -0.51

Mean difference (mCI – mEval) = 4%-points (SD: 12%-points)**

**positive values are more conservative

Pearson correlation = -0.06

Mean difference (mCI – mEval) = 6%-points (SD: 16%-points)**

**positive values are more conservative

Pearson correlation = 0.68

Mean difference (mCI – mEval) = 10%-points (SD: 23%-points)***

***positive values are less conservative
